# Facilitators and Barriers to the Implementation of the HPV VACs (Vaccinate Adolescents Against Cancers) Program: A Consolidated Framework for Implementation Research Analysis

**DOI:** 10.5888/pcd16.180406

**Published:** 2019-07-03

**Authors:** Cam Escoffery, Kara Riehman, Lesley Watson, A. Sandy Priess, Marcie Fisher Borne, Sean Nathaniel Halpin, Carlie Rhiness, Emily Wiggins, Michelle C. Kegler

**Affiliations:** 1 Rollins School of Public Health, Emory University, Atlanta, Georgia; 2 Health Management Associates, Atlanta, Georgia; 3 American Cancer Society, Atlanta, Georgia

## Abstract

**Purpose and Objectives:**

The human papillomavirus (HPV) vaccine is an effective but underused method for preventing multiple cancers, particularly cervical cancer. Although interventions have successfully targeted barriers to HPV vaccine uptake in various clinical settings, few studies have explored their implementation. Our study examines the delivery of the HPV VACs (Vaccinate Adolescents Against Cancer) Program and elicits information on barriers and facilitators to implementation.

**Intervention Approach:**

The VACs Program pilot was a multilevel, evidence-based intervention conducted by the American Cancer Society in 30 federally qualified health centers (FQHCs) in the United States.

**Evaluation Methods:**

We conducted in-depth interviews (N = 32) by telephone with representatives of 9 FQHC partners. We structured the interview guides on Consolidated Framework for Implementation Research (CFIR) domains. We asked about project start-up activities, implementation strategy selection, policy- and practice-level changes, staffing structure, challenges, and key factors leading to project success. At least 2 researchers coded each interview transcript verbatim.

**Results:**

Participants most frequently identified the electronic health record system, training and education, concrete tools and resources, and provider champions as facilitators to implementing HPV VACs. Limited staff resources, challenges of electronic health records, issues with state immunization registries, patient misinformation about vaccines and vaccine stigma, cultural/language barriers, competing priorities, levels of funding, staff buy-in, training needs, and low health literacy were identified as barriers.

**Implications for Public Health:**

Providing appropriate training for FQHC staff members and providers along with technical assistance and facilitation tools were critical for increasing provider confidence in recommending HPV vaccine. Addressing capacity-building and implementation barriers in FQHCs can increase effective implementation of evidence-based interventions to increase HPV vaccination uptake and reduce the burden of future cancers.

**Summary**

**What is already known about this topic?**

The human papillomavirus (HPV) vaccine is an effective but underused method for preventing multiple cancers, particularly cervical cancer. Although many evidence-based interventions exist for increasing HPV vaccine uptake, little is known about the implementation of these interventions.

**What is added by this report?**

Our study identifies facilitators and barriers to delivery of evidence-based interventions for HPV vaccination in federally qualified health centers using an implementation science framework.

**What are the implications for public health practice?**

Training, capacity building, use of electronic health systems, and implementation tools are important factors for successful implementation of evidence-based interventions for HPV vaccination.

## Introduction

About 4 of 5 people in the United States will get human papillomavirus (HPV) at some point in their lives (1). Each year, an estimated 32,500 Americans receive a diagnosis of cancer associated with HPV (2). Through HPV vaccination, about 29,000 cases of cancer could be prevented each year in the United States (2). Despite this opportunity, only 60% of adolescents in the United States initiated HPV vaccination in 2016, and only 43.4% of those aged 13 to 17 were up to date with the HPV series (49.5% for girls; 37.5% for boys), leaving many children exposed to future cancer risk (3,4). The Centers for Disease Control and Prevention estimates that if the HPV vaccine were routinely given with other recommended vaccines, HPV vaccination rates could exceed 90% (5).

Many barriers to HPV vaccination exist, including providers’ ability to give a confident recommendation to vaccinate, parental hesitancy, and missed opportunities to vaccinate (6). A provider recommendation is one of the strongest predictors of HPV vaccination; parents who receive a strong recommendation are more likely to vaccinate their adolescent than those who do not (7–9). HPV vaccination is also influenced by practice factors, such as adequate health information systems that include provider or patient prompts for vaccine administration (10). Interventions to address both provider capacity to deliver an effective recommendation to parents and system challenges have increased HPV vaccination rates in various clinical settings (11). However, few explorations of the delivery of HPV vaccine interventions have been studied qualitatively. The Consolidated Framework for Implementation Research (CFIR) is a theoretical model often used in implementation research to study factors that affect implementation through quantitative or qualitative methods (12). Only 2 qualitative studies have explored barriers (eg, lack of appointment reminders, language) and facilitators (eg, publicity about the vaccine) related to HPV vaccination in health centers across the CFIR domains (13,14). More research is needed to explore the prevalence of these barriers and facilitators across health care settings and populations served.

## Purpose and Objectives

The purpose of this study was to examine the delivery of the HPV VACs (Vaccinate Adolescents against Cancers) Program and to elicit information on barriers and facilitators to implementation related to CFIR domains and constructs. The VACs pilot program was a multilevel intervention conducted by the American Cancer Society (ACS) in 30 federally qualified health centers (FQHCs) in the United States. FQHCs are community-based health care centers that receive funds from the Health Research Services Administration’s Health Center Program to provide primary care services in medically underserved areas. The VACs Program’s goal was to increase HPV vaccination rates among adolescents aged 11 or 12 nationwide. VACs includes work on many levels, from state-level coalition building to clinical interventions. The intervention (hereinafter, “the project”) was a pilot project in 2015 to implement evidence-based quality improvement interventions with FQHC partners nationwide to increase HPV vaccination rates. The project was implemented by ACS Primary Care Systems staff members who collaborated with FQHC systems. These staff members help FQHCs and other primary care system partners implement cancer prevention and early detection efforts. To prepare for the project, ACS Primary Care Systems staff members received 16 hours of intensive training provided by doctoral-level members of our research team with expertise in quality improvement coaching, HPV vaccination science, and evidence-based interventions to increase HPV vaccination rates in the primary care setting. During the pilot, partner FQHCs increased their HPV series initiation rates by an average 15.4% (15).

## Intervention Approach

Thirty FQHC systems implemented the project in 130 clinical or school-based sites. ACS randomly placed the systems into 3 intervention groups, with 10 systems in each group: one group received a $90,000 2-year grant, another group received a $10,000 12-month grant, and another group received training and technical assistance but no funding. Intervention requirements differed somewhat between groups, allowing us to explore the effect of varying funding levels, time frames, and requirements. Details on the selection process are available elsewhere (15).

The intervention included both system-focused components aimed at removing systemic barriers to vaccination and provider-focused components (eg, training, provider prompts, standing orders, assessment and feedback) aimed at improving the quality of providers’ recommendations for the HPV vaccine. Some FQHC systems also implemented patient-focused reminder and recall components. In all 3 groups, FQHC systems worked with ACS partners to educate their staff on HPV vaccination and train providers to make an effective recommendation for the HPV vaccine. All 3 groups were also required to complete a capacity assessment tool, calculate baseline HPV vaccination rates, and modify their electronic health record (EHR) systems to support the project (eg, vaccination reports, provider prompts). Beyond these core strategies, we encouraged the FQHC systems and ACS Primary Care staff members to choose additional strategies for evidence-based interventions, such as provider assessment and feedback, standing orders for HPV vaccination, provider prompts, and patient reminders, on the basis of needs identified during the capacity-building phase. The $90,000 group was required to use at least one additional implementation strategy, but many FQHCs in the other intervention groups did so as well.

## Evaluation Methods

We conducted in-depth interviews by telephone of a sample of FQHC staff members and ACS Primary Care Systems staff members. Nine FQHC partners participated in the interviews: 5 systems funded at $90,000, 2 systems funded at $10,000, and 2 unfunded systems. We selected FQHCs on the basis of several criteria, including not having participated in a previous technical assistance site visit, degree of success in improving rates of HPV vaccination uptake, geographic location, urbanicity, and population served. We purposely chose 5 FQHCs that received $90,000. The evaluation team contacted ACS Primary Care Systems members who were partnering with the selected FQHCs, and these staff members contacted the FQHC to request an interview and ask for a list of participating staff members who would be appropriate for an interview. One FQHC declined to participate, so we selected another FQHC from the same funding group. 

The ACS evaluation team, in partnership with the HPV VACs project team, developed the interview guides. Topics included project start-up activities, implementation strategy selection, policy-level and practice-level changes, staffing structure, project challenges, and key factors leading to project success. Interview guides were structured on CFIR domains: intervention characteristics (key attributes of the intervention), outer setting (contexts outside of the organization), inner setting (structural and cultural processes within the organization), characteristics of individuals (characteristics of the individuals receiving and interacting with the intervention), and implementation process (how and by whom an intervention is delivered) and their associated constructs (14). Questions were similar across FQHCs, although we tailored guides to each respondent type (eg, project champion, nurse).

We conducted 32 interviews from May to August 2016; the number of interviewees per FQHC ranged from 2 to 5. The unit of analysis was the FQHC. Respondents included project champions, nurse coordinators or nurse practitioners overseeing project implementation, quality improvement directors, chief medical officers, and nurses and medical assistants responsible for administering vaccinations. We also conducted interviews with ACS Primary Care Systems staff members working with the 9 FQHCs on the project.

All interviews were digitally recorded and transcribed verbatim. On the basis of the interview guide and CFIR constructs, we developed a provisional codebook of codes and definitions. The Emory research team revised the codebook after reviewing the first few transcripts. Once the Emory research team reached consensus on codes and definitions, 7 team members coded the interview transcripts independently. At least 2 team members coded each transcript, and the team resolved discrepancies. We applied a quasi-deductive approach to data analysis, which emphasized both a deductive and an inductive approach (16). In the first stage, we coded transcripts deductively on the basis of CFIR constructs and definitions, and researchers coded strictly on the basis of the CFIR domains without making inferences from the data. In the second stage, we applied an inductive approach to the first level of codes, seeking to identify subthemes within CFIR domains. We created matrices of key themes, and then we assessed the number of FQHCs identifying each barrier and facilitator. Only barriers and facilitators that were described by 2 or more FQHCs were incorporated into this study. Additionally, we applied a rating system with 2 dimensions: magnitude and valence. “Magnitude” refers to the extent to which the constructs were discussed; we counted the number of times each construct was discussed and tabulated data according to whether 1 respondent noted the construct or 2 or more respondents did so. We further organized the data by funding level (grant of $90,000, grant of $10,000, and technical assistance only). “Valence” refers to the construct’s influence on implementation of the program. We considered valence to be positive (facilitated implementation of the intervention), negative (hindered the implementation), or mixed. We tabulated these data for each construct and further organized by funding level.

## Results

The sample FQHCs represented a mix of urban, rural, and suburban settings; 2 systems had multiple locations in a mix of settings. The number of clinics in each FQHC system ranged from 5 to 22 (Table 1). The number of patients in the eligible age range for HPV vaccination ranged from 661 to 2,777. Common implementation strategies were training (5 of 9 FQHCs trained at least 75% of their staff members) and provider prompts (5 of 9 FQHCs used provider prompts).

**Table 1:** Characteristics of FQHCs (N = 9) Participating in Qualitative Interviews in a Study of Facilitators and Barriers to Implementing the HPV VACs (Vaccinate Adolescents Against Cancers) Program, May–August 2016

FQHC		Outcome		Contextual Factors				Implementation Strategies				
FQHC ID No.	State (No. of Clinics in System)	Baseline HPV Vaccination Initiation Rate, %	Percentage-Point Change in HPV Vaccination Initiation	Funding Level	Urbanicity	Target Patient Population in 2015	Strong EHR Capabilities	75% of Staff Trained	Client Reminders	Provider Prompts	Standing Orders	Provider Assessment and Feedback
1	North Carolina (10)	72.6	23.4	Technical assistance	Urban	904						
2	Maryland (5)	46.4	22.2	Technical assistance	Rural	926	**√**		**√**	**√**		
3	California (5)	73.0	No data	$10,000	Rural	661				√		
4	Maine (13)	52.3	4.2	$10,000	Rural	1,666	**√**	**√**	**√**	**√**	**√**	**√**
5	Florida (6)	64.8	24.9	$90,000	Urban	1,613	**√**	**√**	**√**	**√**	**√**	
6	South Carolina (22)	74.8	−18.7	$90,000	Mixed	2,777		**√**				
7	Alabama (10)	71.2	12.3	$90,000	Mixed	1,541				**√**	**√**	**√**
8	California (10)	15.8	45.4	$90,000	Suburban	1,862		**√**				
9	West Virginia (13)	11.4	3.9	$90,000	Rural	745		**√**				

Abbreviation: **√**, FQHC has characteristic; EHR, electronic health record; FQHC, federally qualified health center; HPV, human papillomavirus.

### Facilitators to implementing HPV VACs

The strongest facilitators, occurring in 4 or more of the FQHCs interviewed, centered on the CFIR domain of intervention process. They were usefulness of EHR systems, ACS staff support, trainings, concrete tools and resources, and provider champions. Examples of themes by CFIR domain and a representative quote follow. 

#### Intervention process

**EHR system (7 FQHCs).** The EHR system was a central focus of the initiative, not only for tracking vaccinations but also for reminding providers. Seven of 9 FQHCs integrated reminders for HPV vaccination into their already established EHR system. A nurse practitioner stated, “We actually already had a prompt built into the EMRs [electronic medical records], so it wasn’t a whole lot of work on our end.”

**ACS staff support (7 FQHCs).** Another key facilitator commonly mentioned was the training, technical assistance, and support offered by ACS staff. A research coordinator commented, “We have a really great ACS staff member, so everything is going good.”

**Provider champions (5 FQHCs).** Engaging with influential people who actively support an intervention can be an important boost to implementation. Designating a provider champion who helped encourage program objectives reinforced HPV vaccination as a priority. In addition to affirming the importance of HPV vaccination, this symbolic appointment helped ensure that education and messaging spread consistently among clinic personnel. An ACS Primary Care Systems staff member shared how a physician champion was instrumental to implementation in her clinic:

It was Dr. [name] who said we’re going to take this and everybody got on board. . . . [H]e’s been a champion from day one. And that makes things easier. If I compare my other two [projects], the other educational grant that I have, I think the key here is that he is a champion and he gets it, and he sees the value and the importance of it. So for me, that has been the key here.

**Training and education (5 FQHCs).** Many FQHCs reported that training from ACS and/or pharmaceutical representatives was essential in ensuring that all team members had the knowledge necessary to implement the project. In particular, this training was important for medical assistants — frontline workers assigned to educate patients about the HPV vaccine who, before the training, often did not have the knowledge necessary to effectively complete this task. Providers expressed greater confidence in the vaccine and their ability to talk with patients about vaccination after the training. A clinical coordinator of pediatrics said the following:

I thought the education was probably one of the most important pieces. I didn’t realize how much some of the staff really didn’t know about it. . . . So I think it was eye opening, especially to some of the medical assistant staff that were kind of on the fence. Personally on the fence with their own children and hard for them to probably make, I would think, a strong recommendation if they are on the fence themselves. So I think that was a really important piece.

**Tools and resources (5 FQHCs).** Along with educating providers, having accessible tools, such as posters highlighting HPV vaccination, was considered a critical tool for successfully increasing HPV vaccination. FQHCs described how having available patient resources helped initiate conversation, and at times these resources would prompt parents to begin the discussion. A nurse practitioner described, “I think the posters have been a nice addition. . . . I think that was helpful in starting a conversation, by the time you walk in the room it’s already — the parent’s already read about it and decided.”

**Written protocols and processes (3 FQHCs).** Having formal processes in place helped with implementation of the VACs program. One health provider remarked, “I think having a strategic plan regarding everyone being on the same plate and doing things the same way in each center because if each center was doing something different, it would be crazy.”

#### Inner setting

The second group of major facilitators related to the inner setting. They represented critical areas such as leadership support, clinical staff support, communications, and teamwork.

**Leadership support (4 FQHCs).** In addition to staff support and teamwork, supportive leadership was important, especially when there were competing demands. Four FQHCs indicated the importance of leadership support for prioritizing the project when other demands might take have taken precedence in daily operations. One quality improvement director stated the following:

Oh, the executive team is very supportive. I report straight to the CEO [chief executive officer] and the chief operations officer. We couldn’t have done it without them because again, I mean working with operations and the CMO [chief medical officer] you have to work as a team in order to roll out a QI [quality improvement] initiative that you may want to. If you don’t have support then projects are not successful.

**Clinical staff support (4 FQHCs). **Four FQHCs acknowledged the importance of active involvement of clinic staff in communicating and promoting awareness among their peer network. For some FQHCs this was a specific “peer coordination team” and for others, a particular nurse or physician took on these roles.

**Communication (4 FQHCs). **Four FQHCs mentioned the importance of effective verbal communication among staff members as a facilitator. Those who described communication as a facilitator appreciated the complexity of communicating with a large group of individuals who each had competing demands and priorities. These FQHCs described identifying effective methods of communication, which could possibly adapt over time given changing needs, as particularly important given the network of providers spread over multiple clinics.

**Teamwork (3 FQHCs).** A pediatrician stated, “I think it’s teamwork. I mean, just someone in charge, tracking the monthly things. That’s the key factor that will help buy success in this project.”

#### Other CFIR domains

Some CFIR domains comprised themes that were mentioned as facilitators by only 2 or 3 FQHCs (Table 2). Related to outer setting, the presence of patient buy-in and creation of partnerships for promotion of the vaccine and vaccination (eg, pharmaceutical companies, schools) emerged as themes. Noted as contributing to program implementation was the fact that the VACs program was compatible and could be integrated easily because some FQHCs had good systems developed for other health projects, and that staff members had knowledge about HPV and the vaccine. 

**Table 2:** Themes of Less Frequently Mentioned^a^ Facilitators or Barriers, Aligned by Domains of the Consolidated Framework for Implementation Research, in Qualitative Interviews in a Study of Implementing the HPV VACs (Vaccinate Adolescents Against Cancers) Program in Federally Qualified Health Centers, May–August 2016

Domain/Theme	No. of FQHCs Noting Barrier	Description of Facilitator or Barrier
**Facilitators**		
**Inner setting**		
Teamwork	3	Although it was important to have a champion to help encourage ongoing support of the project, it was also critical to have team members who were willing to work together to achieve a common goal across the hierarchy. These teams typically consisted of both clinical staff (eg, nurses, medical assistants, physicians) and nonclinicians (eg, quality managers).
**Intervention characteristics**		
Compatibility with other similar projects	3	The VACs project was integrated more easily into FQHCs that had other ongoing projects with similar requirements. These FQHCs were able to add requirements, such as reporting success rates and missed opportunities to clinicians, without the need to establish a new process. This limited the complexity of integrating new activities into existing processes.
**Implementation process**		
Written protocols and processes	3	In addition to human capital, processes were also important to implementation. FQHCs that developed a written plan found this process to be helpful for ensuring step-by-step tasks were completed. Uniformity of procedures, such as when to follow up with patients who are due for vaccination were also useful, particularly for those who had multiple FQHCs in their FQHC system. These plans were typically written in advance of the program being implemented, but they were adaptable, and could be changed to meet shifting requirements.
Previsit planning	3	A key process change discussed in several FQHCs was the identification of patients eligible for HPV vaccination before appointments. This change was particularly helpful for FQHCs that did not already have similar programs in place. These FQHCs found the training in methods for identifying potential HPV vaccine candidates helpful for starting this new process of provider counseling.
FQHC visits	2	FQHC visits by ACS staff were important for “hold[ing] people accountable” to the objectives of the intervention. Additionally, the in-person interaction helped to solidify relationships between ACS representatives and clinic staff members.
**Characteristics of individuals**		
Staff knowledge	2	Staff familiarity and comfort level with HPV vaccine were important facilitators. Staff knowledge of facts of HPV was important in building a general knowledge base for justifying the importance of the vaccine and the scheduled series. This knowledge also extended to building comfort in discussing the vaccine with patients through increased levels of confidence in staff members’ ability to answer patients’ questions.
**Outer setting**		
Families and family buy-in	2	Addressing the wide variety of family needs across different patient populations was critical for meeting the goals of this project. For example, some patient populations had no firsthand knowledge of vaccine-preventable illnesses and so relied on their provider’s knowledge. Providers sometimes stated they had or they would give the vaccine to their own children. Other families had emigrated from locations where vaccine-preventable diseases were prevalent; these first- and second-generation immigrants were very amendable to preventive vaccines because of their recent history.
**Barriers**		
**Process**		
Incomplete program information from ACS	3	Three FQHCs reported receiving incomplete program information from ACS. Without a full understanding of what was required of them, the FQHCs ran into issues with running reports on short timelines and being unable to prepare their staff for what was required of them on the project.
Vaccine supply acquisition	2	Two FQHCs mentioned that they had issues ordering the vaccine and keeping the vaccine in stock. One FQHC recounted the process in which they transitioned from the Gardasil 4 vaccine to the Gardasil 9 vaccine (Gardasil 4 prevents 4 types of HPV and Gardasil 9 prevents 9 types of HPV). Another FQHC reported they had increased the number of HPV vaccinations in their FQHC to the extent that they ran out of the vaccine and had to rush to get more in stock. One director of quality and clinical practice manager recounted, “The financial piece of how we were acquiring vaccines created a little bit of difficulty because since we’re getting it through a 340B program which is a discounted price. And we weren’t dealing directly with the vendor. We weren’t able to initially move away from Gardasil 4 and get the Gardasil 9. So that was kind of a difficult transition since obviously we had to finish those doses of Gardasil 4.”
**Outer setting**		
Patient reach	3	Another issue related to patients was reaching the appropriate patient population and difficulty with having children come to clinic for well-child appointments. At the time of the study, 3 appointments were required to vaccinate against HPV. FQHCs reported higher success rates with the first vaccination, because it aligned with other vaccines that are required for school attendance, but the second and third doses was a problem for this hard-to-reach age group.
Communication between providers and caregivers	3	The quality of communication among people both within the organization and with parents and patients has the potential to affect the implementation process. Three FQHCs had problems coordinating communication among a diverse group of clinician and nonclinicians with various schedules and responsibilities. Communication with parents and patients was also a challenge, particularly given the framing of HPV vaccine as a choice rather than a requirement. One director of quality and risk management stated, “You have to be careful how you present it to the parent as well. Since it’s not required most parents don’t want their child to have it.”
Insurance provider coverage issues	3	Three FQHCs described problems related to billing insurance companies for HPV vaccination. Even though the Centers for Disease Control and Prevention recommends vaccination starting at age 9, some insurance companies will not reimburse for children younger than 11. FQHCs reported that even if they are committed to this project and want to increase vaccine uptake, outside factors such as inconsistent insurance reimbursement hinder their progress.

Abbreviations: ACS, American Cancer Society; FQHC, federally qualified health center; HPV, human papillomavirus.

a The facilitators and barriers described in this table were noted by 2 or 3 FQHCs, whereas the facilitators and barriers described in the text were noted by at least 4 FQHCs.

### Barriers to implementing HPV VACs

All FQHCs reported both barriers and facilitators (Table 2). Several barriers were in all CFIR domains; however, the frequently mentioned barriers were in intervention process, inner setting, and outer settings. Descriptions of the most common barriers by CFIR domain and a representative quote follow. 

#### Intervention process

For the intervention process, 2 FQHCs mentioned difficulties in acquiring the HPV vaccine, and all 9 FQHCs reported difficulties in executing the VACs program. These difficulties revolved around EHRs, staff time, and reaching patients.

**EHR issues (8 FQHCs).** Although EHRs facilitated HPV vaccination through features such as provider reminders, study participants also identified barriers in using their EHR system and barriers in communication between their own EHR systems and other systems. Eight FQHCs mentioned the EHR system as a barrier to full and effective program implementation. Challenges included obtaining information on baseline HPV vaccination completion rates for all 3 doses and switching EHR systems during the project. One director of quality and risk management stated he was not able to use the EHR system to capture data in the project because “we went to a new EHR and the data in the prior EHR that we had did not move over to the new system.”

**Staff resources, including time (8 FQHCs).** Staff resources were cited as a major barrier to implementing the HPV vaccination program in 8 FQHCs. Resources, for the purpose of this study, were defined as references to money, training, education, materials, physical space, and time. Most staff resources barriers revolved around the idea that taking providers out of the clinic for trainings slowed down productivity and the clinic’s ability to provide care to their patients. Numerous clinics also reported that taking time for training and other intervention processes would cost the clinic in terms of lost revenue. A family nurse practitioner stated, “Any time you have to pull medical assistants or receptionists, front end staff out of practice, you’re minimizing or making a challenge for people to get in for care, which is the opposite of what we’re trying to do.”

#### Inner setting

Barriers in the inner setting reflected relative priority of the VACs program, readiness for implementation through staff buy-in, available resources, communications, and need for training.

**Competing priorities (7 FQHCs).** Seven FQHCs reported competing priorities that negatively affected project implementation. In particular, fitting the project into the existing workflow, given the demands of acute walk-in patients who required immediate assistance, was a challenge. One registered nurse panel manager stated, “Keeping in the forefront with other duties that the medical assistants and providers have; they’re constantly being inundated with things that have to be taken care of in the system for documenting and so on.”

**Staff buy-in (5 FQHCs).** Although a system was in place whereby clinical staff communicated with their peers to get buy-in, this communication did not happen for some staff members. Five FQHCs stated that not all staff members were committed to project implementation. Front desk staff members and providers showed occasional resistance to the program because of the major changes in protocol the project required, resulting in slower implementation of the project. One director of quality and risk management stated, “Staff are not assisting me in scheduling them for their second and third HPV, so that has to be a learning curve and education for the staff, and we are doing much better to get their commitment to that now.”

**Training needs (5 FQHCs)**. Five FQHCs reported barriers to implementation that focused on training needs. Formal education was a major piece of this intervention, and although formal education facilitated implementation, some FQHCs also had ideas on how to improve uptake of key information. Some FQHCs faced problems disseminating information to clinical and nonclinical staff using terminology and examples that were appropriate across their roles. One ACS primary care manager stated, “We’re trying to engage their scheduling staff, their front desk staff who aren’t clinical, don’t have a clinical background to understand the message and to help engage clients. A lot of the training . . . [is] more clinically focused.”

**Communication (3 FQHCs).** Communication challenges were reported; an ACS staff member stated, “With the HPV project, . . . we had these HPV champions, the provider champion, and these MAs [medical assistants] per clinic site to help facilitate the projects. It’s a lot harder to get them together to meet as a group to talk about how the project is coming along.”

#### Outer setting

Barriers in the outer setting domain reflected patient-related factors, such as misinformation, language barriers, and low levels of literacy. In addition, linkage to the state immunization registries was often noted as challenging.

**Patient misinformation and vaccine stigma (8 FQHCs). **Providers reported difficulties recommending the HPV vaccine to patients when vaccine stigma and patient/parent misinformation surrounding HPV and its vaccine existed. Many parents had either general antivaccine sentiments — whereby they refused all optional vaccines — or they had negative opinions of the HPV vaccine specifically. Many parents believed the vaccine promoted sexual activity in their children or they thought children only needed the vaccine if they were sexually active. One medical assistant trainer stated, “They feel that, oh my child isn’t sexually active, so I don’t need that.”

**Cultural or language barriers (5 FQHCs).** Cultural and language issues were mentioned as a barrier to implementation in 5 FQHCs. Frequently, FQHCs required more skills and materials to accommodate their non–English-speaking patients than their English-speaking patients. One director of quality and clinical practice management stated, “The only problem that we do have is that we don’t have anything in Creole. So that makes it complicated.”

**Low health literacy** (**4 FQHCs**). Four FQHCs said that increased resources were necessary for educating patients with low health literacy. Many patients with low health literacy had very little experience with primary care, and thus were not accustomed to receiving preventive care. A chief medical officer stated, “Prior to the Affordable Care Act a lot of these people had no insurance. . . . [T]here is also a cultural deficit of health literacy just because there’s never been any access to health care.”

**State registry issues** (**6 FQHCs**). Six FQHCs faced issues with their state immunization registry. Most states have an online immunization information system where providers are required to report all immunizations administered at their FQHC. Ideally, an EHR system communicates with the state registry to prevent the need for separate data entry into both systems and reconciliation between the 2 systems. Many FQHCs did not have bidirectional communication between their EHR system and state immunization registry, resulting in more work for providers and staff members to ensure both systems are accurate and up-to-date. An ACS representative stated, “The immunization, for our state, it’s not bidirectional with the immunization registry. So it is incumbent upon the FQHC to keep that updated.”

**Level of ACS funding/cost of program** (**5 FQHCs**). Five FQHCs mentioned that the cost of the program and the level of funding from ACS were incompatible, resulting in concerns that the funding provided was not adequate for the effort required. One of these FQHCs received $90,000, 2 received $10,000, and 2 received only technical assistance. One nurse practitioner stated, “I think we ended up with a $10,000 reward, which doesn’t really cover the costs as you can imagine for all the work that we do.”

A smaller set of FQHCs mentioned patient reach, communication between provider and caregivers, insurance provider coverage issues, incomplete program information from ACS, and barriers that centered on vaccine acquisition (Table 2).

### Distribution of themes within CFIR domains

When we charted how barriers and facilitators were distributed across CFIR domains and constructs, we found that the FQHCs reported barriers or facilitators in 2 of 8 possible constructs for intervention characteristics, 3 of 4 possible constructs for outer setting, 3 of 5 possible constructs for inner setting, 6 of 8 for process, and 1 of 5 for individual characteristics (Figure). For example, facilitators for outer setting constructs relate to patient needs and resources (family buy-in) and cosmopolitanism (ie, collaborating with ACS in incorporating evidence-based interventions into their practices). Implementation facilitators were concentrated in the inner setting and process domains, whereas barriers were distributed throughout the range of domains, except for Individual Characteristics. We found no domains in which all 9 FQHCs reported facilitators to implementation; 9 reported barriers across 2 domains: patient needs and resources (outer setting) and readiness for implementation (inner setting). For example, many barriers were related to the construct of patient needs and resources, including misinformation or hesitancy among patients and caregivers, language barriers of families served, and external policies. CFIR constructs generally were representative across sites; most were mentioned across at least 2 sites (all but 2 themes) (Tables 3 and 4). The construct named most often (by 7 FQHCs) as a facilitator was “executing,” in the process domain. Some factors had both positive and negative effects on implementation, particularly in the domains of intervention process, inner setting, and outer setting (Table 5).

**Figure:**
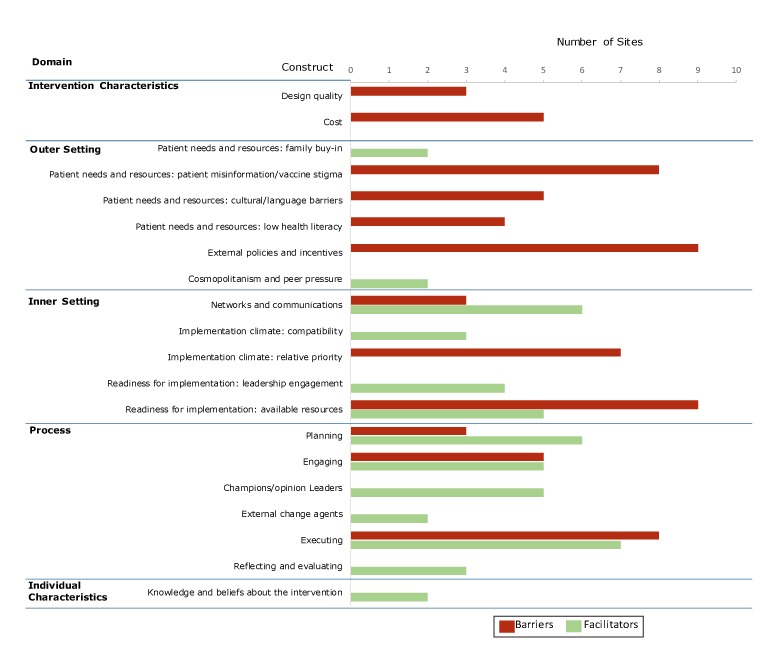
Major themes of barriers and facilitators to implementing the HPV VACs (Vaccinate Adolescents Against Cancers) Program across domains of the Consolidated Framework for Implementation Research (CFIR), May–August 2016. This figure does not show all possible constructs, because the federally qualified health centers participating in the study did not report barriers or facilitators for every construct.

**Table:** 

CFIR Constructs	Barrier	Facilitator
**Intervention characteristics**		
Design quality	3	0
Cost	5	0
**Outer setting**		
Patient needs and resources: family buy-in	0	2
Patient needs and resources: patient misinformation/vaccine stigma	8	0
Patient needs and resources: cultural/language barriers	5	0
Patient needs and resources: low health literacy	4	0
External policies and incentives	9	0
Cosmopolitanism and peer pressure	0	2
**Inner setting**		
Networks and communications	3	6
Implementation climate: compatibility	0	3
Implementation climate: relative priority	7	0
Readiness for implementation: leadership engagement	0	4
Readiness for implementation: available resources	9	5
**Process**		
Planning	3	6
Engaging	5	5
Champions/opinion leaders	0	5
External change agents	0	2
Executing	8	7
Reflecting and evaluating	0	3
**Individual characteristics**		
Knowledge and beliefs about the intervention	0	2

**Table 3:** Magnitude^a^ of Facilitators by Constructs of Consolidated Framework for Implementation Research and Level of Funding, Study of Implementing the HPV VACs (Vaccinate Adolescents Against Cancers) Program, May–August 2016

Facilitator	Received $90,000					Received $10,000		Received Technical Assistance Only	
	5	6	7	8	9	3	4	2	1
**Intervention characteristics**									
Compatibility with other similar projects	**●**							**●**	
**Intervention staff**									
Staff knowledge						**●**		**●**	
**Intervention process**									
Trainings and education			**●**	**●**		**●**	**■**	**■**	
Tools and resources			**●**	**●**			**●**	**●**	**●**
Written protocols and processes			**●**	**●**	**●**				
ACS staff support	**■**	**■**	**■**		**●**		**●**	**●**	**●**
EHR system	**■**		**●**	**■**	**■**		**●**	**■**	**●**
Quality improvement team	**■**								
Previsit planning			**●**			**●**			**●**
Site visits			**●**						**●**
Provider champions	**●**		**●**				**●**	**●**	**●**
**Inner setting**									
Leadership support					**●**	**●**	**●**	**●**	
Clinic staff support			**●**	**●**			**■**	**■**	**●**
Communication				**■**	**●**			**●**	**●**
Teamwork			**●**				**●**		**●**
**Outer setting**									
Patient needs: families/family buy-in			**●**				**●**		
Cosmopolitanism: other partnerships (pharmaceutical companies/schools)	**●**				**■**				

Abbreviations: ●, 1 Participant noted facilitator; ■, ≥2 Participants noted facilitator; ACS, American Cancer Society; EHR, electronic health record; FQHC, federally qualified health center; HPV, human papillomavirus.

a “Magnitude” refers to the extent to which the constructs were discussed. Numbers in column headings refer to the FQHC identification number. Thirty FQHC systems implemented the HPV VACs project. The systems were randomly placed into 3 intervention groups, with 10 systems in each group: one group received a $90,000 2-year grant, another group received a $10,000 12-month grant, and another group received training and technical assistance but no funding. Nine FQHCs were selected to participate in qualitative interviews.

**Table 4:** Magnitude^a^ of Barriers by Constructs of Consolidated Framework for Implementation Research and Level of Funding, Study of Implementing the HPV VACs (Vaccinate Adolescents Against Cancers) Program, May–August 2016

Barrier	Received $90,000					Received $10,000		Received Technical Assistance Only	
	5	6	7	8	9	3	4	2	1
**Intervention characteristics**									
Incomplete program info from ACS				●		■			●
**Intervention staff**									
Inconsistent implementation between providers	●								●
**Intervention process**									
Vaccine acquisition	●			●					
EHR issues	●	●	■	●	■	●	●		■
Staff resources and time	■		■	■	●	■	■	■	■
Patient reach					●		●	●	
**Inner setting**									
Program incompatibility	●		●			●			●
Communication	●		●			■			
Staff buy-in	●		●		●		●		■
Competing priorities	●	●	●	●			●	●	■
Training needs	●	●		■		●	■		
Level of ACS funding/cost of program	■					●	●	●	●
**Outer setting**									
Low health literacy	●		●	●				●	
Immigrant population	●							●	
Cultural barriers/language barriers	■	●	●	●				●	
Time restrictions for patients	■								
Patient misinformation/ vaccine stigma	■		●	■	●	●	■	●	●
Insurance provider coverage issues	●		■					●	
State registry issues	■		■		●		●	●	■

Abbreviations: ●, 1 FQHC noted barrier; ■, ≥2 FQHCs noted barrier; ACS, American Cancer Society; EHR, electronic health record; FQHC, federally qualified health center; HPV, human papillomavirus.

a “Magnitude” refers to the extent to which the constructs were discussed. Numbers in column headings refer to the FQHC identification number. Thirty FQHC systems implemented the HPV VACs project. The systems were randomly placed into 3 intervention groups, with 10 systems in each group: one group received a $90,000 2-year grant, another group received a $10,000 12-month grant, and another group received training and technical assistance but no funding. Nine FQHCs were selected to participate in qualitative interviews.

**Table 5:** Valence^a^ of Constructs of Consolidated Framework for Implementation Research and Level of Funding, Study of Implementing the HPV VACs (Vaccinate Adolescents Against Cancers) Program, May–August 2016

Construct	Received $90,000					Received $10,000		Received Technical Assistance Only	
	5	6	7	8	9	3	4	2	1
**Intervention characteristic**									
Evidence of strength	0	0	0	−	0	−	0	0	−
**Intervention staff**									
Knowledge	−	0	0	0	0	+	0	+	−
**Intervention process**									
Planning: vaccine acquisition and previsit planning	0	0	+	0	0	+	0	0	+
Executing	+/−	+/−	+/−	+/−	+/−	−	+/−	+/−	+/−
Champions (providers)	+	0	+	0	0	0	+	+	+
External change agents (ACS staff)	+	+	+	0	+	0	+	+	+
**Inner setting**									
Compatibility	+/−	0	−	+	0	−	0	0	−
Network and communication	−	0	+/−	+	+	−	+	+	+
Implementation climate: staff buy-in	−	0	+/−	+	−	0	+/−	+	+/−
Relative priority: competing priorities	−	−	−	−	0	0	−	−	−
Leadership engagement	0	0	0	0	+	+	+	+	0
Access to knowledge: training needs	−	−	0	−	0	−	−	0	0
Available resources: level of ACS funding/cost of program	−	0	0	0	0	−	−	−	−
**Outer setting**									
Patient needs	−	−	+/−	−	0	0	+	−	0
Policies and mandates: insurance provider coverage issues	−	0	−	0	0	0	0	−	0
Cosmopolitanism	+/−	0	−	0	+/−	0	−	−	−

Abbreviations: +, positive effect on implementation; −, negative effect on implementation. +/−, mixed effect on implementation, “0”=theme not mentioned in the interview; ACS, American Cancer Society; EHR, electronic health record; FQHC, federally qualified health center; HPV, human papillomavirus.

a “Valence” refers to the construct’s influence on implementation of the program. We considered valence to be positive (facilitated implementation of the intervention), negative (hindered the implementation), or mixed. Numbers in column headings refer to the FQHC identification number. Thirty FQHC systems implemented the HPV VACs project. The systems were randomly placed into 3 intervention groups, with 10 systems in each group: one group received a $90,000 2-year grant, another group received a $10,000 12-month grant, and another group received training and technical assistance but no funding. Nine FQHCs were selected to participate in qualitative interviews.

## Implications for Public Health

Our study identified CFIR-related barriers and facilitators to implementing the HPV VACs Program. The most salient facilitators aligned with the intervention process and inner setting, a finding supported by other implementation science research, indicating that organizations should consider the implementation process and organizational context when adopting evidence-based interventions (17). A recent qualitative study of a pediatric consortium found many facilitators related to CFIR domains of inner setting, intervention staff, and intervention process (14). Our findings show the importance of early planning, engaging staff members and champions, and planning for implementation during the intervention process. The primary facilitators were the use of EHRs and training (intervention process) and education for providers (inner setting). The EHR system is critical to various parts of HPV vaccination services, including documenting vaccination, tracking reminders for the subsequent dose, and generating reports on adolescents who are eligible and those who are vaccinated and reports on missed opportunities (18). Future implementation should also consider the need to provide adequate support to health systems over time about the importance and use of their EHR system to prompt clinic staff to offer a vaccination, document vaccinations, follow up on series completion, and track their health system’s progress on vaccination. Consultation and education from a technical assistance provider, a strategy used in several HPV interventions (19–21), was identified as an important component of successful implementation in our study. Training and skill-building activities are needed to promote provider self-efficacy to counsel parents about vaccination. Building the capacity for alerts or reminders for parents and facilitating communication about vaccine series completion with state immunization registries also are foundational in implementing HPV promotion programs in health centers.

Our study also found that unfunded FQHCs used similar implementation strategies (eg, training, technical assistance, facilitation) and had similar outcomes to funded FQHCs (22), suggesting that ACS staff support or committed FQHC staff members, rather than funding, was the key to project success. These concerted efforts to facilitate CFIR factors related to the execution of the program (intervention process) were critical elements. Other research has found parallel results and reported a relationship between training of program implementers and tools and adoption of evidence-based practices (17,22). Furthermore, a review of interventions to improve physician learning and practice found that educational materials, outreach visits, opinion leaders, and reminders were moderately effective strategies (23). Studies have demonstrated that use of EHR alerts for health care providers alone or in combination with other strategies improves HPV vaccine completion (21,24). Not surprisingly, a strong theme in our study was the use of EHRs to alert providers to recommend vaccination to unvaccinated teenagers. A quantitative study also found that EHRs were both a facilitator and a barrier, crucial to calculating vaccination rates and reminding providers and patients but also presenting challenges in data quality and reporting (13).

In contrast, barriers for implementing evidence-based interventions (eg, staff resources) were concentrated in the domains of implementation process, inner setting, and outer setting. Our finding of a critical mass of barriers (ie, high magnitude) in the outer setting demonstrates the importance of understanding patient issues about the vaccine and social and external influences that affect community awareness and vaccine delivery. The use of CFIR to elucidate themes that have a positive effect on implementation (ie, positive valence) is important to successful implementation of evidence-based interventions and inform potential solutions to address barriers. Some factors had positive and negative effects on implementation, and it is important to have qualitative methods for implementation studies to elicit information on these salient factors.

Perceived barriers are important to address because they can lead to missed opportunities to vaccinate (20,25). FQHCs reported inner setting constructs (staff resources/time and competing priorities) were major limitations to implementing HPV vaccine programs. Likewise, outer setting variables were challenges to implementation. Other key barriers were the ability to communicate bidirectionally with the state immunization registry and patient needs related to language and literacy. These patient-related factors (parental knowledge and antivaccine sentiment) are frequently reported as critical barriers (6,26). Subsequently, health care providers reported vaccine safety concerns among parents and not having adequate time to discuss these concerns during visits (18,27,28). However, providers’ recommendations of the vaccine and higher-quality recommendations are associated with vaccine initiation and series completion (29). Potential tools are materials for providers’ office or parental education (eg, fact sheets) through the Centers for Disease Control and Prevention (2) and the American Academy of Pediatrics HPV Champion Toolkit (30), which assist with practice changes to increase vaccination rates. Finally, another strategy is to assess parental concerns through surveys (31).

In a recent systematic review of interventions designed to increase HPV vaccination, only 11 of 34 interventions focused on provider- or system-based interventions; researchers called for more research for interventions to promote implementation of effective strategies (32). Others have called for more provider/physician interventions to promote HPV vaccine uptake (10). Future research should evaluate multiple components of interventions, such as this initiative, on increasing the initiation and completion of HPV vaccine series among adolescents and young adults. The FQHCs in our study used various implementation strategies. Future evaluations can assess which implementation strategies or combination of strategies could increase HPV vaccination rates (33,34). Additional implementation science studies could examine how CFIR is operationalized in practice and validate our themes mapped as CFIR constructs around barriers and facilitators. Use of an implementation theoretical framework such as CFIR contributes to a broader understanding of contextual barriers and facilitators. Future implementation study can use theory to inform a deeper understanding of factors that affect implementation (35,36). Additional exploration of the use of CFIR constructs in implementation research has been recommended, particularly on how constructs may affect implementation and outcomes (37).

Our study reports facilitators and barriers identified by 9 FQHCs that received varying levels of funding and may be different from other FQHCs or health centers. The study evaluated a 3-shot vaccine regimen; however, recommendations have since changed to 2 scheduled doses 6 months apart (38). Despite this change, our results remain applicable because of the need for an initial vaccination and a follow-up.

Our study provides important insights into barriers and facilitators experienced by clinics stratified by CFIR domains and providers when promoting HPV vaccination in community settings. Themes of capacity building such as training and technical assistance, presence of supports and organizational champions, and key processes such as EHR infrastructure for provider reminders are salient. Chung et al noted the challenges of making practice changes and the importance of considering practice-specific issues when supporting vaccination efforts (39). Furthermore, training and tools exist to help bolster the self-efficacy of providers and to counsel vaccine-uninformed or -hesitant parents. Future implementation should also consider the need to provide adequate support to health systems over time to integrate provider reminders into EHRs and promote provider self-efficacy to counsel parents about vaccination. It is important to understand which factors have shaped the success or failure of implementations of evidence-based interventions for HPV vaccination in health centers that reach low-income populations, such as FQHCs. This information can optimize future implementations of effective HPV vaccine strategies in this context and reduce the burden of future cancers.
